# Unanticipated Side Effects of Stratospheric Albedo Modification Proposals Due to Aerosol Composition and Phase

**DOI:** 10.1038/s41598-019-53595-3

**Published:** 2019-12-11

**Authors:** Daniel J. Cziczo, Martin J. Wolf, Blaž Gasparini, Steffen Münch, Ulrike Lohmann

**Affiliations:** 1 Department of Earth, Atmospheric and Planetary Sciences, Massachusetts Institute of Technology, 77 Massachusetts Avenue, Cambridge, Massachusetts 02139 United States; 2 Department of Civil and Environmental Engineering, Massachusetts Institute of Technology, 77 Massachusetts Avenue, Cambridge, Massachusetts 02139 United States; 3 Department of Earth, Atmospheric and Planetary Sciences, Purdue University, 550 Stadium Mall Drive, West Lafayette, Indiana 47906 United States; 4 Institute for Atmospheric and Climate Science, ETH Zürich, Universitaetstrasse 16, Zurich, 8092 Switzerland; 5 Department of Atmospheric Sciences, University of Washington, 408 ATG, Box 351640, Seattle, Washington 98195 United States

**Keywords:** Atmospheric chemistry, Climate-change mitigation, Atmospheric chemistry

## Abstract

The Earth has now warmed ~1.0 °C since the period 1850–1900, due in large part to the anthropogenic addition of greenhouse gases to the atmosphere. Most strategies to address this warming have called for a reduction of emissions and, often, accompanying removal of greenhouse gases. Other proposals suggest masking the increased radiative forcing by an increase in particles and/or clouds to increase scattering of incoming solar radiation. Two related recent proposals have suggested addition of calcite particles to the stratosphere, which one model suggests may enhance ozone. Here we show that the interaction of calcite with acidic materials in the stratosphere results in a more complex aerosol than has been previously considered, including aqueous and hydrate phases that can lead to ozone loss. Our study suggests particle addition to the stratosphere could also perturb global radiative balance by affecting high altitude cloud formation and properties. Experimental and modeling results suggest particles will act as the nucleation sites for polar stratospheric cloud ice and, after sedimentation into the troposphere, impact cirrus clouds in the absence of other efficient ice nucleating particles. These results show that an overly simplistic set of assumptions regarding intentional particle emissions to the atmosphere can lead to incorrect estimates of the radiative effect and fail to identify unintended consequences.

## Introduction

It has been established that the anthropogenic emissions of greenhouse gases have warmed the planet by ~1.0 °C since pre-industrial times^1,2^. There have been proposals to intentionally alter the atmospheric abundance of greenhouse gases both to use their warming potential to raise temperature for the benefit of colder climates^3^ and, more recently, to lessen the detrimental effects of increased global temperature^4^. The latter is termed ‘carbon capture and storage’ and falls within the concept of ‘geoengineering’, commonly defined as the intentional manipulation of planetary processes for a desired climatic effect.

Since at least the 1960’s there have been proposals to mask the increased radiative forcing associated with anthropogenic greenhouse gases by increasing planetary albedo^5–8^. Land and ocean albedo enhancement and, more recently, the addition of light-scattering particles to the atmosphere and/or manipulation of cloud properties have been proposed^2^. Highlighting the uncertainty surrounding these actions, a recent report by the National Academy of Sciences^2^ suggests such processes should be described as ‘climate interventions’ rather than ‘climate engineering’ or ‘climate management,’ terms which imply a level of certainty that is not supported by experimental evidence. These albedo modification strategies do not address the other effects of greenhouse gases, such as ocean acidification^2^, and are predicted to have significant side effects, including changes in plant growth, precipitation, stratospheric heating and ozone loss and reduced concentrated solar power generation^9,10^. Increased scattering of solar radiation, termed shortwave radiation (SW), the cornerstone of albedo modification, also has side effects such as changes in precipitation and atmospheric chemistry^6,10^.

**Figure 1:**
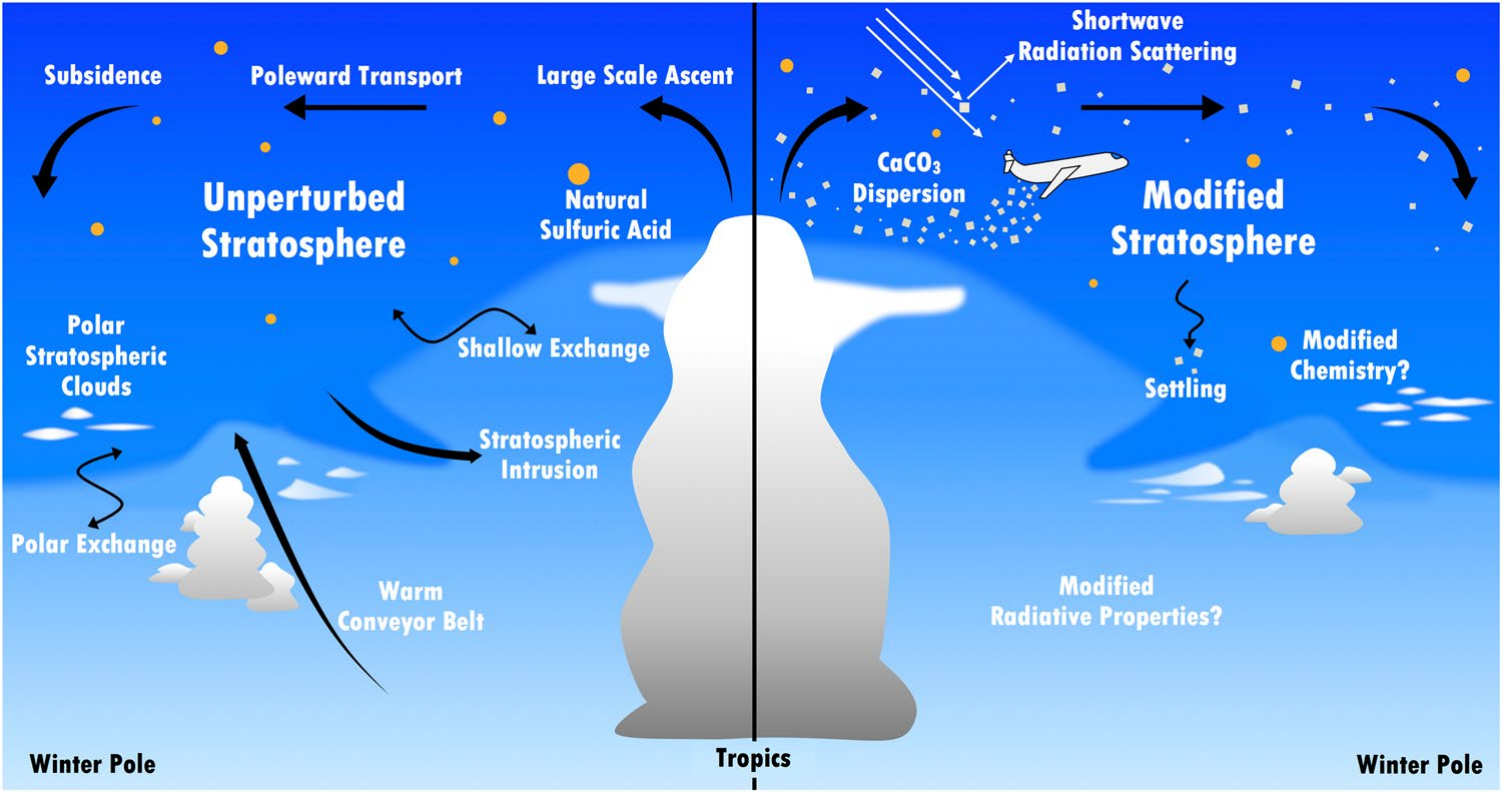
Conceptual model of the tropopause region in an unperturbed (left) and modified case (right). The color delineates the stratosphere from troposphere. In the unperturbed case, there is a general circulation from the equator to wintertime pole with subsidence into the polar vortex. Sedimentation, shallow exchange and stratospheric intrusions/folds move material downward across the tropopause whereas warm conveyer belts and convection can move material upward. In the modified case, particles are injected into the stratosphere in an attempt to reflect more sunlight before it reaches the surface. In this case the material placed in the stratosphere can impact stratospheric chemistry by the presence of new surface area. The different radiative balance in the stratosphere can affect the troposphere and sedimented particles may impact cirrus ice and polar stratospheric cloud formation.

A number of proposals for climate intervention have suggested augmentation of the natural stratospheric aqueous sulfuric acid aerosol layer^7^. Figure 1 shows this concept in relation to an unperturbed atmosphere. Volcanic eruptions have been observed to inject sulfuric acid precursors and water vapor into the stratosphere, leading to enhanced particle formation^11^. Mixing and sedimentation processes result in particle lifetimes on the order of a few years^12,13^. Volcanic enhancement of the concentration of light-scattering particles in the stratosphere causes increased scattering of SW, and lower global temperatures, for up to a few years^11^. Volcanic effects are therefore transient, and not analogous to albedo modification proposals, which require a sustained and increasing input to mask a continually increasing concentration of greenhouse gases.

The increase in particles after volcanic eruptions has been observed to lead to a depletion of ozone in the stratospheric layer that absorbs ultraviolet SW before it reaches the surface^11,14^. Two acids, HNO_3_ and HCl, act as stratospheric reservoirs of nitrogen and chlorine radicals (termed NO_x_ and ClO_x_, respectively) that catalytically destroy ozone^15^. Particles in the stratosphere, natural or otherwise, can act as sites for heterogeneous chemistry involving these ozone-destroying radicals. Reactivity varies with particle phase: aqueous solutions, hydrates and ices generally have higher reactivity than anhydrous surfaces^14^. Proposals for albedo modification suggesting augmenting sulfate aerosols would therefore lead to a destruction of ozone^2,11,14^. There have been several comprehensive studies of the intricacies of stratospheric ozone depletion by particles naturally or anthropogenically added to the stratosphere. These consider polar and lower latitudes^2,15^, dynamics^16^, and the amount of injected material^2,17^.

Some recent albedo modification proposals have suggested addition of light scattering but less chemically reactive (with respect to ozone depletion) particles such as alumina^5^ and calcite^5,6^. Calcite (CaCO_3_) addition has been suggested since it could scatter SW and might reduce stratospheric aerosol acidity and sequester NO_x_ and ClO_x_, leading to increased ozone^6^:

1 $${{\rm{CaCO}}}_{3({\rm{s}})}+{{\rm{H}}}_{2}{{\rm{SO}}}_{4({\rm{g}},\mathrm{aq})}-\, > {{\rm{CaSO}}}_{4}+{{\rm{H}}}_{2}{{\rm{O}}}_{({\rm{g}},{\rm{l}})}+{{\rm{CO}}}_{2({\rm{g}})}$$

2 $${{\rm{CaCO}}}_{3({\rm{s}})}+{\rm{2}}\,{{\rm{HNO}}}_{3({\rm{g}},\mathrm{aq})}-\, > {\rm{Ca}}{({{\rm{NO}}}_{3})}_{2}+{{\rm{H}}}_{2}{{\rm{O}}}_{({\rm{g}},{\rm{l}})}+{{\rm{CO}}}_{2({\rm{g}})}$$

3 $${{\rm{CaCO}}}_{3({\rm{s}})}+2\,{{\rm{HCl}}}_{({\rm{g}})}-\, > {{\rm{CaCl}}}_{2}+{{\rm{H}}}_{2}{{\rm{O}}}_{({\rm{g}},{\rm{l}})}+{{\rm{CO}}}_{2({\rm{g}})}$$

The underlying assumption in reactions (1)–(3) is that anhydrous salts are produced and that their surfaces are less reactive than aqueous, hydrate and ice surfaces^6^. Moreover, eventual particle sedimentation would effectively remove part of the stratospheric NO_x_ and ClO_x_ burden. Keith *et al*.^6^ considered an addition of 2.1 and 5.6 Tg calcite per year in the form of 275 nm radius particles, resulting in Ca(NO_3_)_2_, CaSO_4_ and CaCl_2_ due to reaction with nitric, sulfuric and hydrochloric acid, respectively. This order corresponds to the assumed production due to the vapor pressure of each species: production of CaCl_2_ is least favorable due to the high vapor pressure and low abundance of HCl. The estimated radiative impact ranged from 1–2 W/m^2^ between the cases. Based on an assumption of all products being anhydrous and inactive, a 3.8% increase in stratospheric ozone was estimated for the 2.1 Tg case^6^.

**Figure 2:**
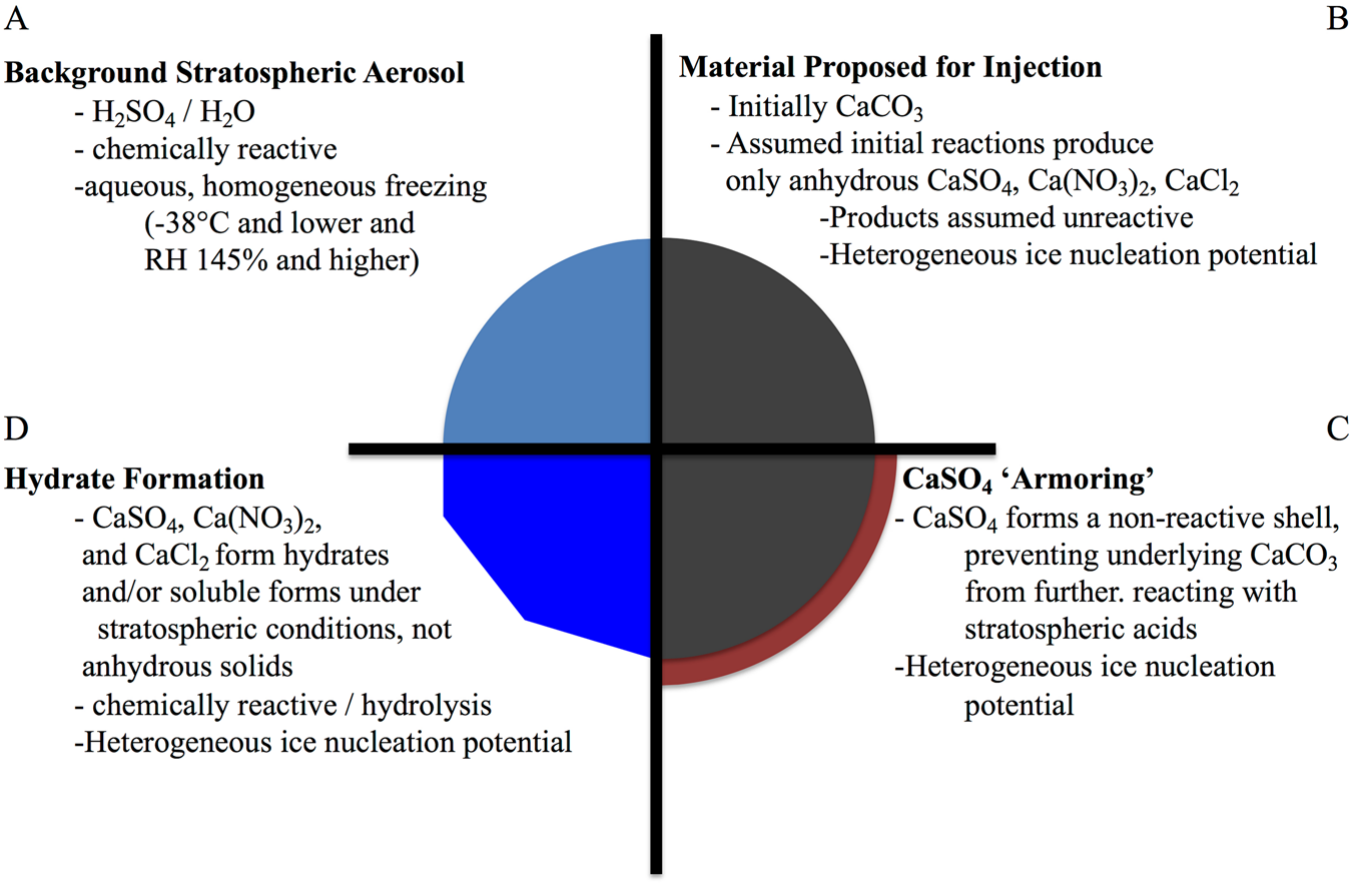
The natural background stratosphere contains aqueous H_2_SO_4_ particles which act as sites for reactions that destroy ozone (Panel A). Proposals for injection of additional aqueous H_2_SO_4_ particles would scatter more solar radiation but promote additional ozone loss^2^. Aqueous H_2_SO_4_ particles do not nucleate ice heterogeneously, instead requiring temperatures below −38 °C and RH with respect to ice in excess of 145%^30^. Recent proposals suggest addition of scattering materials that are less reactive, such as CaCO_3_ (Panel B)^6^. It has been assumed that CaCO_3_ will fully react with stratospheric acids to produce similarly unreactive anhydrous salts^6^. The literature does not support this assumption since sulfuric acid produces an unreactive CaSO_4_ ‘armor’ on CaCO_3_ upon reaction with H_2_SO_4_ (Panel C)^18^. Regardless of the acid-CaCO_3_ reaction, the ultimate product under stratospheric conditions is not anhydrous but instead reactive hydrates and soluble salts (Panel D)^20,25,26^. Unlike aqueous H_2_SO_4_, CaCO_3_ and the solid and hydrate products can nucleate ice heterogeneously (Fig. 3), thereby introducing effective ice nucleating particles, and impacting cloud formation, to the stratosphere and troposphere after sedimentation. Injected particle composition will evolve over time and single particles will most likely form a mixture of these.

Reactions (1)–(3), although chemically balanced, do not account for the correct form of the products under stratospheric conditions. The basis for Eq. (1) is that the ubiquitous stratospheric aqueous sulfuric acid layer, or sulfuric acid vapor, will react with the injected calcite particles via coagulation or uptake, respectively, and that all reactions will proceed until the calcite is converted (Fig. 2). Full conversion is inconsistent with the literature since CaSO_4_ forms a surface layer that acts as ‘armor’ that prevents further reaction; this reaction has been extensively studied for use of limestone mitigation of acid mine drainage^18^. Any incomplete conversion of CaCO_3_ results in a smaller ozone increase than suggested by Keith *et al*.^6^.

CaSO_4_ exists in several forms not realized in Eq. (1) that have been extensively studied for industrial purposes. These include CaSO_4_ dihydrate (gypsum), hemihydrate (CaSO_4_•1/2H_2_O), dihydrate (CaSO_4_•2H_2_O) and both soluble and insoluble anhydrites^19^. Laboratories studies show production of the soluble anhydrite CaSO_4_ at stratospheric temperatures (<200 K) and low relative humidity (RH), transitioning to the dihydrate at ~40% RH^20^. Room temperature studies show that the hemihydrate is produced between these two phases, at ~20%, and the hexahydrate forms at 70–80% RH^19^. Ozone depleting reactions of these compounds have not been studied, but other hydrates, such as nitric acid trihydrate, effectively promote ozone loss^14^.

Keith *et al*.^6^ only discuss formation of an anhydrite (the difference between the soluble and insoluble forms is not noted), but at the mean RH with respect to liquid water of the lower stratosphere in the extratropics (~12 km altitude) of ~12%^21^ aqueous particles are the thermodynamically favorable form. The focus of most stratospheric aerosol augmentation studies to increase global albedo is on a mid-stratospheric layer, between ~20–25 km^2 ^^4–6^ where the mixing ratio of water vapor is 3–7 parts per million by volume (ppmv)^22,23^. There is a strong water vapor gradient from the tropopause to mid-stratosphere, with a decreasing RH as altitude increases. Values of 40% RH are not uncommon in the lower stratosphere, decreasing to 3% above 20 km^21,24^. Particles are more likely to exist in anhydrous and lower hydrate forms at higher altitudes and as higher hydrates or in aqueous states closer to the tropopause. The exact phase state will depend on the specific particle type and water vapor and temperature profile. Overall, our analysis suggests that current assumptions of CaSO_4_ particle phase are oversimplified and sulfate sequestration and ozone depletion impacts need to be reconsidered.

Ca(NO_3_)_2_, similarly, is only found in its anhydrous form at very low (<7%) relative humidity (RH)^25^. Ca(NO_3_)_2_ deliquesces to di-, tri- and tetrahydrate forms^26^. Liu *et al*.^25^ showed that both Ca(NO_3_)_2_ and mixed Ca(NO_3_)_2_/CaCO_3_ particles are hydrated above 7% RH, and therefore would be active for ozone depleting reactions well below the average humidity of the stratosphere. CaCl_2_ has a similar behavior and its phase is known from studies of Martian materials^27^. Above 13% RH at 223 K it exists as di-, tetra- and hexahydrate with the last transition at 80% RH at 223K^27^. Instead of considering reactions (1)–(3) leading to different anhydrous and unreactive particles, the more consistent concept would be a menagerie of salts, hydrates and unreacted calcite in different proportions, possibly on the same particle. To our knowledge, the reactivity of such a complex aerosol has not been quantified.

**Figure 3:**
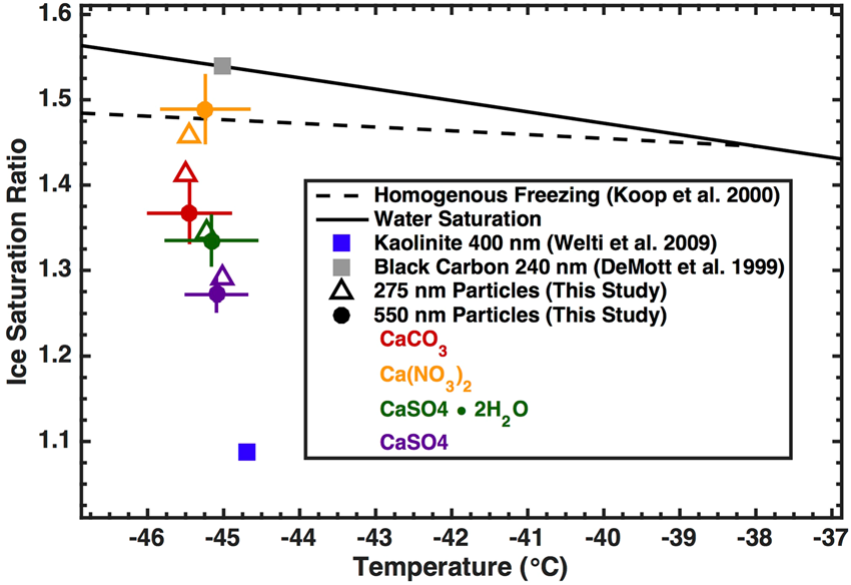
The temperature and relative humidity required for ice nucleation by the particles proposed for addition to the stratosphere and the products expected from acid reactions. Water saturation (solid line) and homogeneous freezing (dashed line)^30^ are shown for reference. Conditions required for ice nucleation are shown for 275 and 550 nm diameter particles. For comparison, an effective tropospheric ice nucleating particle (INP), kaolinite mineral dust, and an ineffective INP, elemental carbon (EC), are shown. The proposed injection material, CaCO_3_, and the anhydrous and hydrate forms of CaSO_4_ were found to nucleate ice with moderate effectiveness. Calcium nitrate, incorrectly assumed to be an anhydrous salt by Keith *et al*.^6^, is not an effective INP, since it is either a hydrate or an aqueous solution under these conditions.

Regardless of their ultimate composition, stratospheric particles eventually sediment through the tropopause; downward transport of stratospheric air is particularly strong in the polar vortex in winter due to diabatic descent (Fig. 1)^12,13^. Once in the troposphere, particles interact with water vapor and can act as cloud nuclei, forming droplets or ice crystals^28^. The formation of droplets is understood from Köhler theory^29^ and droplet producing particles are termed cloud condensation nuclei (CCN). Ice nucleation is more complex. Ice nucleates homogeneously from droplets, such as aqueous H_2_SO_4_ (Fig. 2), at a humidity near liquid water saturation and a supercooling of ~40 K below the equilibrium freezing temperature^30^. Ice can form at higher temperatures and lower RH heterogeneously, empirically determined, on ice nucleation particles (INPs)^28^. The efficacy of a particle to act as an INP depends on the material, the size and surface properties^28^. Using an ice cloud chamber, we have produced relevantly sized CaCO_3_, CaSO_4_ and Ca(NO_3_)_2_ from anhydrous materials and hydrates and exposed them to temperatures and RHs commonly found in the upper troposphere in order to determine their potential as INPs (Fig. 3; Supplementary Information contains a discussion of the methods). Calcite, which is expected to remain a solid particle, and anhydrous and the dihydrate of CaSO_4_, act as moderately effective INPs. Ca(NO_3_)_2_, which exists as higher order hydrates and in solution under these conditions, is only observed to nucleate ice homogeneously. This finding also reinforces the reactivity of Ca(NO_3_)_2_ for ozone depleting reactions. The ability of a fraction of injected particles to act as INPs is important in two regimes: upper tropospheric cirrus ice and polar stratospheric clouds. The former case is further discussed in the next paragraphs. The role of heterogeneous nucleation on polar stratospheric clouds, sites on which ozone-depleting reactions occur^14^, is not resolved. Thus, the impact on cloud formation and ozone depletion from addition of anthropogenic INPs to this region of the atmosphere is currently unknown.

**Figure 4:**
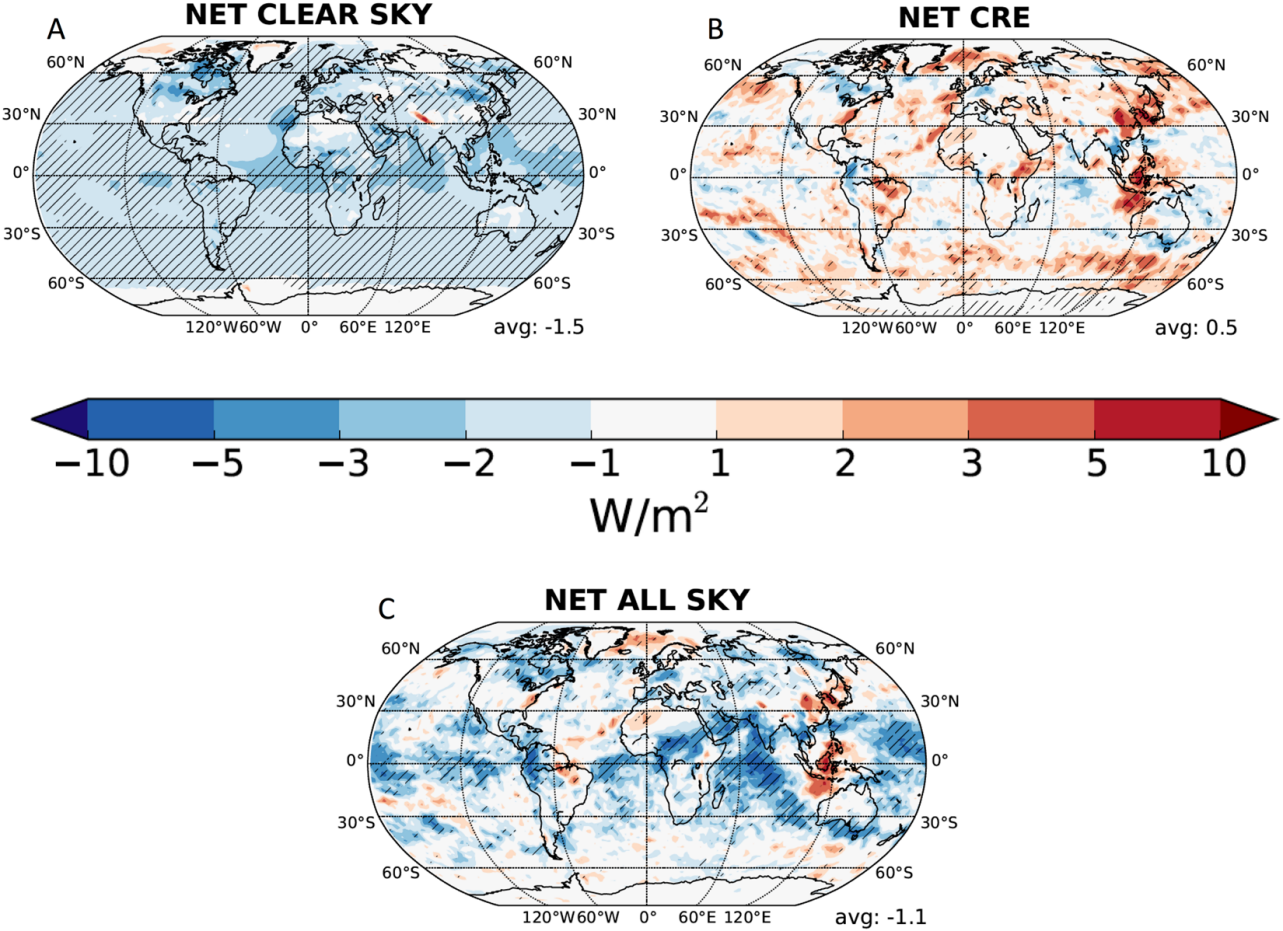
Annual mean changes in radiative fluxes for years 4–10 of a 10 year simulation (see Supplementary Materials for details). The net clear sky radiative effect at the top of the atmosphere between the proposed maintenance of 5.6 Tg CaCO_3_ in the stratosphere and an unperturbed atmosphere is shown in Panel A where hatching marks areas with changes above a 95% confidence level. The net cooling is −1.5 W/m^2^, comparable to the −2 W/m^2^ suggested by Keith *et al*.^6^. A 33% reduction (0.5 W/m^2^) is found for the net cloud radiative effect (CRE) (Panel B) due to less SW radiation available for tropospheric clouds to scatter (i.e., a “cloud shielding” effect). The net full sky radiative effect of the injection is thus only −0.9 W/m^2^ (Panel C). The balance can be attributed to changes in cloud properties by the sedimenting aerosol and interannual variability (see Supplementary Materials for further discussion).

In order to estimate the impact of injection of calcite into the stratosphere we use a general circulation model which can simulate aerosol transport, evolution, and radiative effects as well as aerosol-cloud interactions in both liquid and ice clouds. We simulate a continuous stratospheric injection of calcite particles with a modal radius of 275 nm with the calcite refractive indices consistent with Keith *et al*.^6^. The calcite burden required ~3 model years to equilibrate at about 5 Mt, consistent with a particle lifetime of just under 1 year (Fig. S1). The simulated radiative forcing from calcite aerosols alone is −1.5 W/m^2^ averaged for years 4–10 of the simulation (Fig. 4, panel A). This result is comparable to the value of −2 W/m^2^ reported by Keith *et al*.^6^; the variance is due to differences in aerosol treatment (modal vs. sectional aerosol schemes) and the interactive simulation of stratosphere-to-troposphere transport. Sedimentation processes are normally well resolved in models, however, coarse vertical resolution in the stratosphere compared to thin aerosol or cloud layers may lead to numerical diffusion and be a limitation for properly resolving circulation. This can affect correctly simulating the residence time of particles in the stratosphere.

The decreased SW radiation flux that reaches tropospheric clouds reduces their ability to scatter solar radiation, implying a positive (warming) cloud radiative effect of 0.5 W/m^2^ (Fig. 4, panel B) with the net radiative effect of the calcite injection at −1.1 W/m^2^ (Fig. 4, panel C). It is unclear if Keith *et al*.^6^ considered this “cloud shielding” effect but it has been previously shown for sulfate and more complex injection simulations^31,32^. Our simulations also allow sedimentation of particles across the tropopause and their ability to either form new cloud particles or shift their formation mechanisms based on the laboratory measurements^33^. The treatment of ice cloud formation mechanism (homogeneous versus heterogeneous nucleation) varies between models and has been shown to result in substantial radiative difference^33–35^. Our model simulations only show small changes in cirrus clouds in case of abundant natural INPs and larger changes if natural INPs are rare (Supplementary Materials). These results show that a full consideration of the chemical, physical and radiative impacts of albedo modification proposals is necessary to provide an understanding of the impact on the planet.

## Methods

Particles were produced from aqueous solutions, anhydrous and hydrate crystals, depending on their predicted phase state at tropopause conditions. Ice nucleation onset was determined within a liter-sized cloud chamber^36^. Also known as an ‘ice cloud chamber’, tropopause conditions of temperature and relative humidity at which cirrus clouds form can be controlled to test INP properties. For the global climate simulations, the ECHAM-HAM general circulation model was used. ECHAM-HAM includes a two-moment aerosol scheme, capable of simulating aerosol emissions, growth, coagulation, and sinks and a two-moment cloud microphysics scheme with prognostic equations for cloud liquid and ice, suitable for simulations of aerosol-cloud interactions^37,38^.

The Supplementary Information contains a full discussion of the methods.

## Supplementary information

Supplementary Information
