# Supplementary material for: Unanticipated Side Effects of Stratospheric Albedo Modification Proposals Due to Aerosol Composition and Phase
Source: Sci Rep. 2019 Dec 11;9:18825. doi: 10.1038/s41598-019-53595-3 (PMC6906325; doi:10.1038/s41598-019-53595-3)
Supplement: Supplementary file 1 — Supplementary Information [file 41598_2019_53595_MOESM1_ESM.pdf]

Unanticipated Side Effects of Stratospheric Albedo Modification Proposals Due to Aerosol Composition and Phase : Supplementary Information

Daniel J. Cziczo[1,2,3]*, Martin J. Wolf[1], Blaž Gasparini[4,5], Steffen Münch[4] and Ulrike Lohmann[4]

[1]Department of Earth, Atmospheric and Planetary Sciences, Massachusetts Institute of Technology, 77 Massachusetts Avenue, Cambridge, Massachusetts 02139, United States

[2]Department of Civil and Environmental Engineering, Massachusetts Institute of Technology, 77 Massachusetts Avenue, Cambridge, Massachusetts 02139, United States

[3]Department of Earth, Atmospheric and Planetary Sciences, Purdue University, 550 Stadium Mall Drive, West Lafayette, Indiana, 47906, United States

[4]Institute for Atmospheric and Climate Science, ETH Zürich, CHN O11 Universitaetstrasse 16, Zurich, 8092, Switzerland

[5]Department of Atmospheric Sciences, University of Washington, 408 ATG, Box 351640, Seattle, Washington 98195, United States

*e-mail: djcziczo@purdue.edu

Methods

Spectrometer for Ice Nuclei (SPIN)

The SPIN instrument (Droplet Measurement Technologies, Boulder, CO) is a commercially available ice cloud chamber that has been discussed in detail previously[1,2]. Briefly, SPIN consists of parallel flat plates which are temperature controlled and separated by a 10.0 mm gap. Prior to experiments, each plate is coated with 1.0 mm of ice. Aerosol particles to be investigated enter the instrument in a laminar flow of 1.0 liters per minute (lpm). Particles are constrained to the centerline of the gap by sheath flows of 4.5 lpm along each wall (9 lpm total). The temperature and saturation ratio that the particles experience is controlled by the temperature of the walls which can be varied over the course of an experiment. An integrated optical particle counter determines light scattering from particles and ice crystals exiting SPIN, thereby determining the threshold humidity required for ice nucleation as a function of temperature. For the experiments here, a typical upper tropospheric temperature[3] below the homogeneous freezing threshold was used (-43 - -47° C).

Aerosol particles were generated by either atomizing a solution of $Ca(NO_3)_2$ or by dry dispersal of ground samples of $CaCO_3$, $CaSO_4$, and $CaSO_4{\bullet}2H_2O$ (Aldrich) in an agitated flask generator[4]. Aqueous $Ca(NO_3)_2$ particles were dried to <20% relative humidity by passing the aerosol stream through a diffusion dryer. Particles were size selected using a Brechtel Manufacturing Inc. Differential Mobility Analyzer[5] (DMA, Model 2002) using a sheath to sample flow ratio of 10:1.

Modeling

Simulation setup and ECHAM-HAM model

The simulations were performed with the ECHAM-HAM general circulation model coupled to the HAM2 aerosol and cloud microphysics module[6]. The simulations were run at 1.875° x 1.875° horizontal resolution with 47 levels in vertical extending up to 0.01 hPa. The evaluation of the performance of mean climate, climate variability, and climate sensitivity of the underlying base model can be found in Stevens et al. (2013). ECHAM-HAM has been described in terms of the aerosol and cloud scheme and evaluated in Neubauer et al.[6] The aerosols are divided by size in 4 separate lognormal modes: nucleation (radius < 0.005 μm), Aitken (0.005 μm < radius < 0.05 μm), accumulation (0.05 μm < radius < 0.5 μm) and coarse mode (radius > 0.5 μm). We inject the calcite particles into the accumulation mode, which has a predetermined modal width of 1.59. The distribution of the injected particles therefore spans a broader size range than the one in Keith et al.[7], explaining part of the difference in the clear-sky radiative forcing between the two studies. We injected calcite aerosols in a continuous manner in model levels 15 – 18, spanning the altitude range of 19.5 – 25.7 km, in a zonally uniform latitudinal band between 30°S and 30°N. In the simulation the injected aerosols are assumed to be microphysically inert and not take up sulphuric acid or coagulate with each other or with other aerosols.

A fixed sea surface temperature model setup was used and the first 3 years and 3 months of simulation were discarded (i.e., October 1999 – December 2002) to allow sufficient time for equilibration of the calcite aerosol burden for a 5.6 Mt injection strategy (Figure S1). Years 3-9 of the simulations are used in the analysis of radiative fluxes, microphysical changes, and anomalies. The black vertical line in Figure S1 represents the beginning of the period over which the averages were computed.

Cloud radiative effects for the 5.6 Mt simulations

The decomposition of the total cloud radiative effects into their shortwave (SW) and longwave (LW) components reveals that most of the net change derives from the SW, while the LW component does not have large areas of significant changes. This pattern is true for both simulations with (Figure S2) and without calcite (Figure S3).

Cirrus cloud freezing and calcite aerosol-cirrus cloud interactions

The ECHAM-HAM model considers the competition between homogeneous freezing of sulfuric acid solution droplets, heterogeneous freezing on mineral dust particles, and vapor deposition on pre-existing ice crystals[8,9]. In order to study effects of calcite aerosols on cirrus clouds, we consider calcite aerosol to be active as deposition nucleation ice nuclei. The ice nucleation threshold, a Relatively Humidity with respect to ice (RH_{ice}) of 135%, was determined in the laboratory experiments. This onset threshold lies above that of mineral dust (110% RH_{ice} for temperatures colder than -53°C and 120% RH_{ice} at warmer temperatures), and dust particles immersed in sulfuric acid droplets (130% RH_{ice}). Heterogeneous ice nucleation dominates in the ECHAM-HAM model. A vertically integrated representation of the relative importance of the homogeneous ice crystal nucleation (Figure S4) for the reference model (no calcite injection) confirms the relatively minor fraction of homogeneous nucleation in the ECHAM-HAM model. In ECHAM-HAM cirrus clouds form predominantly by homogeneous nucleation only over mountainous regions and in the tropopause layer[8]. Note that different models use different ice nucleation schemes and our result is not expected to be universal.

Warm cirrus[10] originate mainly from the detrained and other liquid-origin ice crystal sources[11]. These cirrus clouds are radiatively more important than colder cirrus due to their

greater ice crystal number concentrations, liquid water content, and optical thickness[10]. This was shown to be in good agreement with satellite observations[11]. The microphysical formation of such liquid-origin cirrus cannot be modified through direct effects of heterogeneous ice nucleation. The modeled cirrus clouds show a good agreement with CALIPSO data in terms of median ice properties but show a higher extinction compared to observations[10].

Following the injection of calcite aerosols for an injection rate of 5.6 Mt/year there are a few areas with a decrease in the homogeneous freezing ratio (Figure 5A,B). The robustness of this findings is investigated with a sensitivity test in which we decrease the fraction of natural ice nucleating particles (INP) to 1%, which increases the simulated fraction of homogeneous cirrus clouds. This result would be e.g. applicable to models that form less cirrus ice heterogeneously. In this case, large areas are susceptible to cirrus seeding by calcite or other particles (Figure S5C,D).

Supplementary Figures

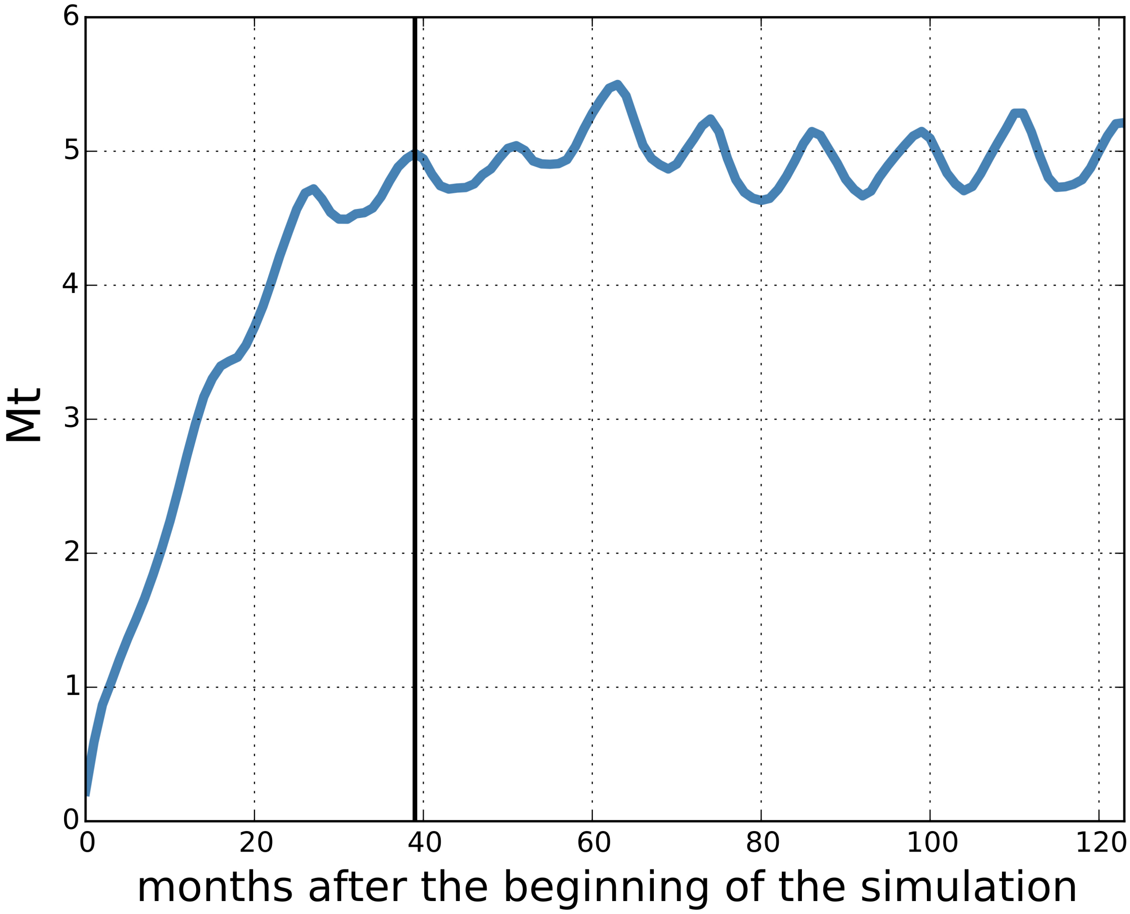

Figure S1 Simulated calcite burden (Mt) versus time after the start of the simulation.

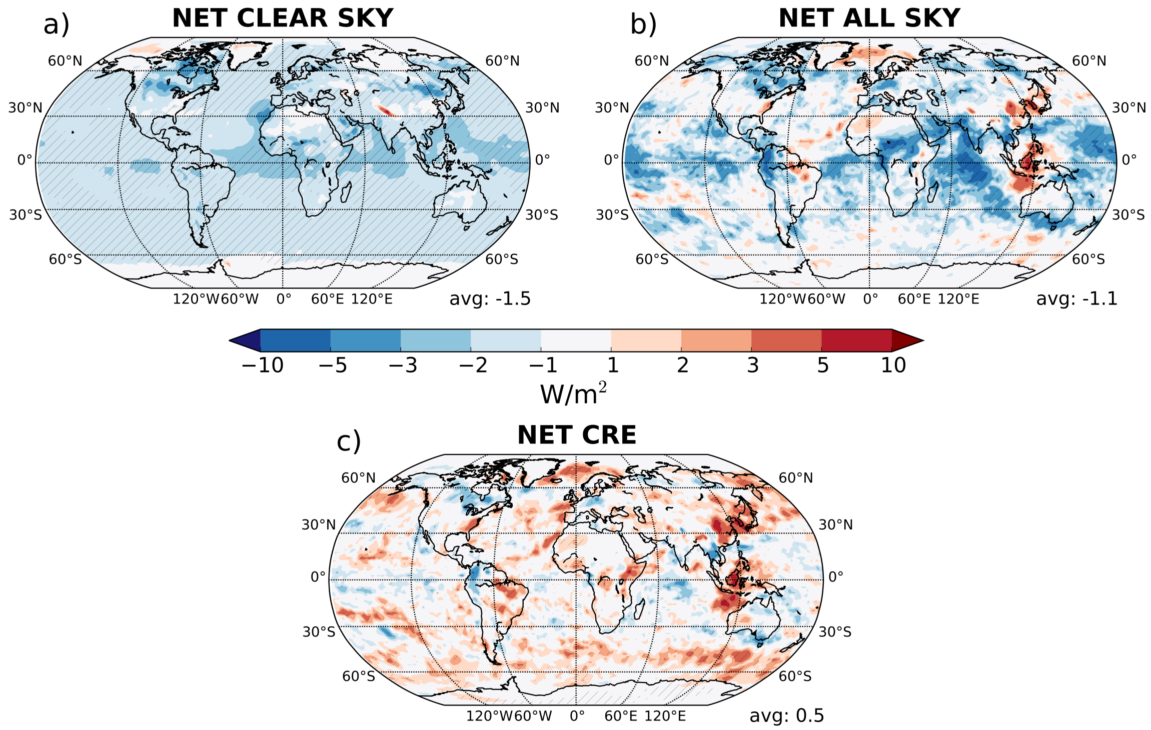

Figure S2 Top of atmosphere (TOA) radiative effects for a stratosphere with a 5.6 Mt calcite burden where no perturbation of cirrus clouds is allowed. The effect of the stratospheric aerosol in the absence of other cloud effects is the net clear sky (panel a) forcing. The net all sky (panel b) forcing includes the reduction in scattering by tropospheric clouds due to the SW scattering increase in the stratosphere. The net cloud radiative effect (CRE) (panel c) reveals that the reduction in scattering is a net warming of 0.5 W/m^2. Hatching indicates changes at the 95% confidence level.

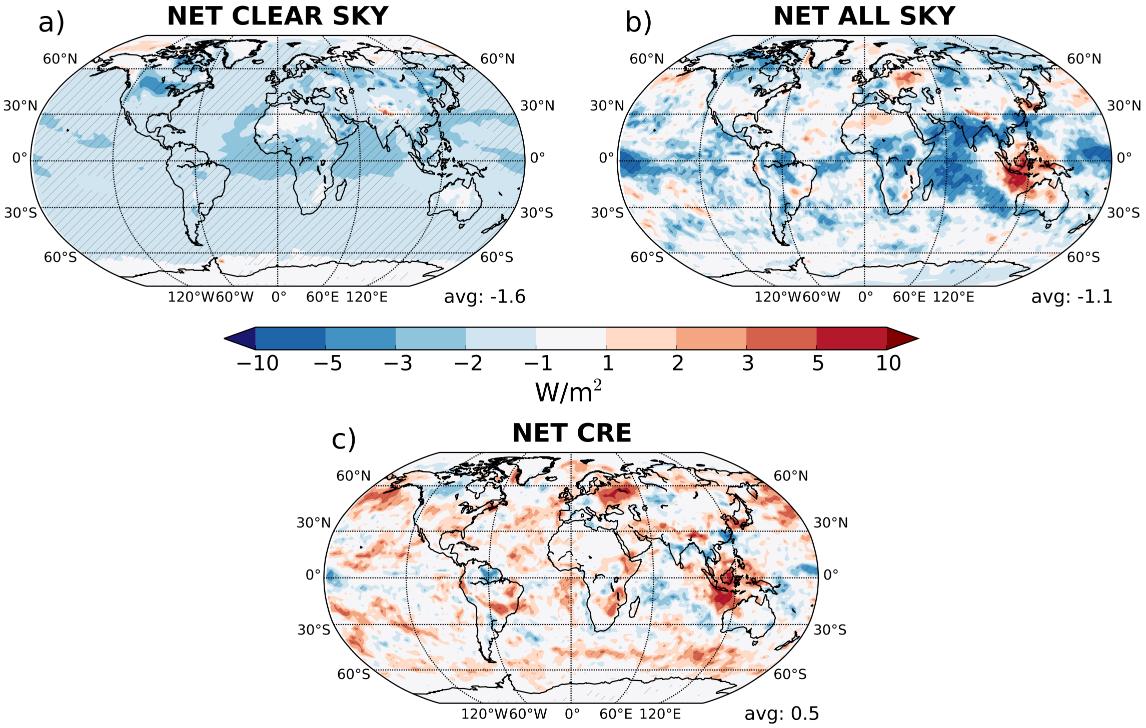

Figure S3 Analogous to Figure S2 where cirrus clouds are allowed to be microphysically impacted by sedimented particles. The radiative forcing of the clouds and interannual variability is 0.1 W/m^2.

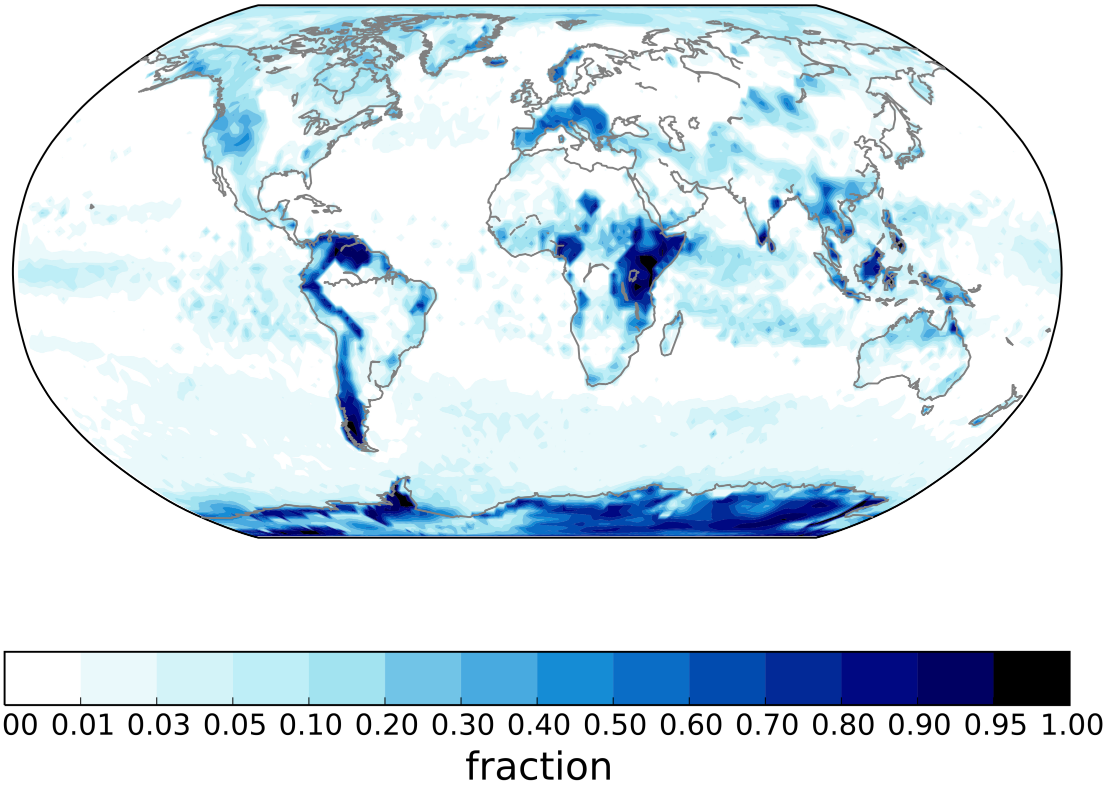

Figure S4 Vertically integrated fraction of ice nucleated homogeneously in the reference version of the ECHAM-HAM model. Note that homogeneous nucleation is predominantly associated with mountain ranges and associated large updrafts.

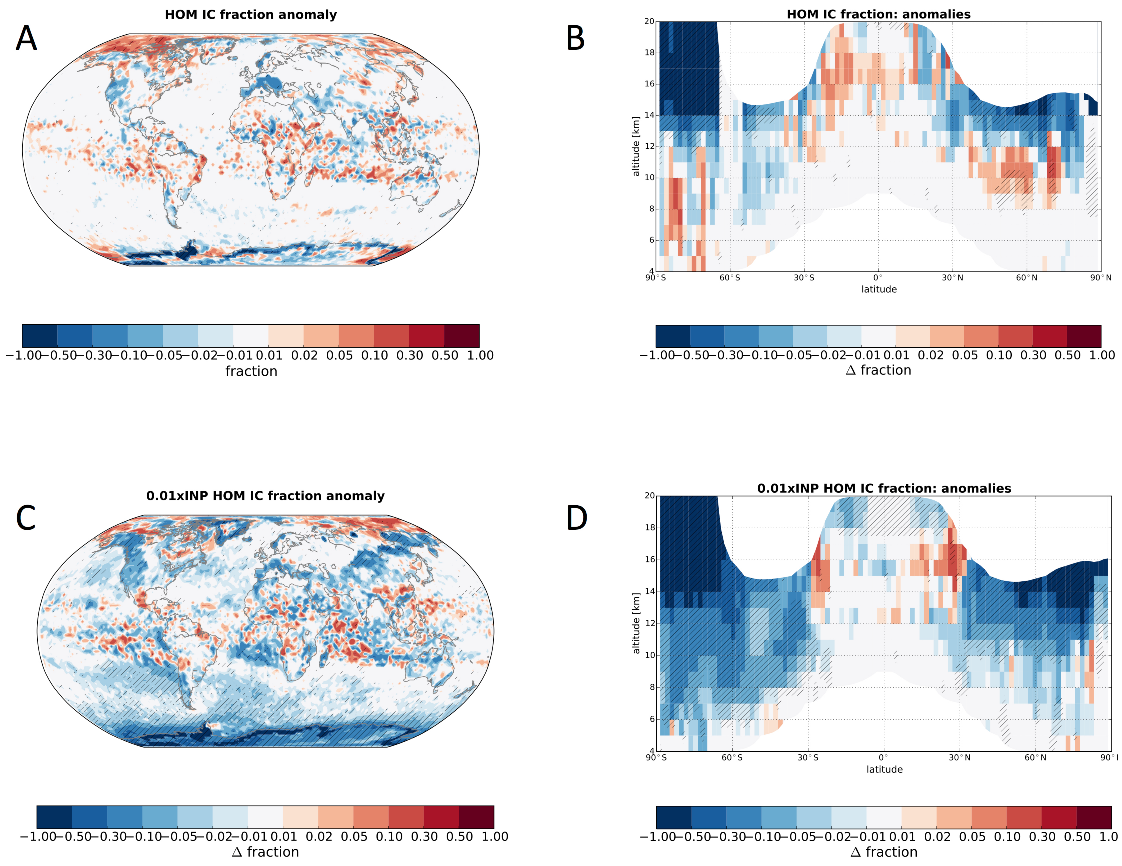

Figure S5 Panel A: Vertically integrated change in homogeneous ice nucleation due to sedimenting particles from stratospheric injection. Hatched areas indicate changes at the 95% confidence level. Panel B: Zonal average of change in homogeneous ice nucleation due to sedimenting particles from stratospheric injection. Panel C: Sensitivity test of vertically integrated change in homogeneous ice nucleation due to sedimenting particles from stratospheric injection with 1% of the ice nucleating particles in the reference ECHAM-HAM simulation. Panel D: Zonal average of changes in homogeneous ice nucleation for the sensitivity test of vertically integrated change in homogeneous ice nucleation due to sedimenting particles from stratospheric injection with 1% of the ice nucleating particles in the reference ECHAM-HAM simulation.

References

1. Garimella, S. *et al.* The SPectrometer for Ice Nuclei (SPIN): an instrument to investigate ice nucleation. *Atmos. Meas. Tech.* **9**, 2781-2795 (2016).
2. Garimella, S. *et al.* Uncertainty in counting ice nucleating particles with continuous flow diffusion chambers. *Atmos. Chem. Phys.* **17**, 10855-10864 (2017).
3. Gierens, K., Schumann, U., Helten, M., Smit, H. & Marenco, A. A distribution law for relative humidity in the upper troposphere and lower stratosphere derived from three years of MOZAIC measurements. *Ann. Geophys.* **17**, 1218-1226 (1999).
4. Garmiella, S., Huang, Y.-W., Seewald, J. S. & Cziczo, D. J. Cloud condensation nucleus activity comparison of dry- and wet-generated mineral dust aerosol: the significance of soluble material. *Atmos. Chem. Phys*., **14**, 6003–6019 (2014).
5. Wiedensohler, A. *et al.* Mobility Particle Size Spectrometers: Harmonization of Technical Standards and Data Structure to Facilitate High Quality Long-Term Observations of Atmospheric Particle Number Size Distributions. Atmos. Meas. Tech. **5**, 657–685 (2012).
6. Neubauer, D., Lohmann, U., Hoose, C. & Frontoso, M. G. Impact of the representation of marine stratocumulus clouds on the anthropogenic aerosol effect, Atmos. Chem. Phys. **14**, 11997–12022 (2014)
7. Keith, D. W., Weisenstein, D. K., Dykema, J. A. & Keutsch, F. N. Stratospheric solar geoengineering without ozone loss. *Proc. Natl. Acad. Sci.* **113**, 14910-14914 (2016).
8. Gasparini, B. & Lohmann, U. Why cirrus cloud seeding cannot substantially cool the planet. *J. Geophys. Res.* **121**, 4877-4893 (2016).
9. Kuebbeler, M., Lohmann, U. & Feichter, J. Effects of stratospheric sulfate aerosol geo-engineering on cirrus clouds. *Geophys. Res. Lett.* **39**, L23803 (2012).
10. Gasparini, B. *et al.* Cirrus Cloud Properties as Seen by the CALIPSO Satellite and ECHAM-HAM Global Climate Model. *J.Clim.,* **31**, 5 (2018).
11. Gasparini, B. *et al.* Is increasing ice crystal sedimentation velocity in geoengineering simulations a good proxy for cirrus cloud seeding? *Atmos. Chem. Phys.,* **17**, 4871-4885 (2017).
